# A Distributed Framework for the Study of Organizational Cognition in Meetings

**DOI:** 10.3389/fpsyg.2022.769007

**Published:** 2022-05-18

**Authors:** Astrid Jensen, Davide Secchi, Thomas Wiben Jensen

**Affiliations:** ^1^Department of Language and Communication, Faculty of Humanities, University of Southern Denmark, Slagelse, Denmark; ^2^Department of Language and Communication, Faculty of Humanities, University of Southern Denmark, Odense, Denmark

**Keywords:** distributed cognition, ecological cognition, organizational cognition, complex adaptive systems, agent-based modeling, multimodal interaction analysis (MMIA)

## Abstract

This paper proposes an analytical framework for the analysis of organizational cognition that borrows from distributed and ecological cognition. In so doing, we take a case study featuring a decision on the topic of agreeing on a set point in the agenda of a meeting. It is through the analysis of a few minutes of video-recording used in the case that enables us to demonstrate the power of applying distributed and ecological cognition to organizing processes. Cognitive mechanism, resources, and processes are identified within this combined framework. Mechanisms are described as “socio-material” (CM1)—where “people” and “artifacts” are the related cognitive resources—and as “conceptual” (CM2)—with “group” identity, “topic” understanding, meaning of “procedures,” and perception of “time” as resources. Processes are defined as “coupling,” “de-coupling,” and “un-coupled” depending on the type of relation in place. Finally, the paper presents an agent-based computational simulation to demonstrate the potentials of operationalizing this approach.

## Introduction

In this paper, we wish to propose an analytical framework for the analysis of *organizational cognition* by focusing on how cognitive processes emerge when individuals use each other and the environment to make sense and interact during a work meeting. To this end, we draw on elements from an ecological paradigm (Hutchins, 2010; Gibson, 2014) and on the perspective of distributed cognition (e.g., Hutchins, 1995; Hollan et al., 2000).

In so doing, we take a case study featuring a group of people agreeing on a point in the agenda of a meeting. It is through the analysis of a few minutes of video-recording used in the case that enables us to demonstrate our approach to analyzing *organizational cognition*, accomplished as a joint organizational practice in an interplay between agents, situations, relations and environment. By focusing on an internal meeting in a Danish subsidiary of a large multinational company, we identify how people use each other and the environment to make sense, understand others and coordinate interaction.

The vantage point in distributed and ecological cognition rests on a break with the tradition of considering the human brain as the only component of cognitive processes while focusing on the system of exploited cognitive resources (e.g., language, body, emotions, artifacts, norms, etc.) that, together with the brain, enable or disable those processes. Though we are conscious about important distinctions in the way in which various strands of cognitive science describe how cognitive processes are enacted, distributed or extended by items outside our brains and bodies (e.g., Clark and Chalmers, 1998; Clark, 2008; Hurley, 2010; Menary, 2010; Kirchhoff, 2012; Gallagher, 2013; Slors, 2019, 2020), the focus on distributed and ecological cognition emanates from a shared assumption that cognitive processes are distributed across parts of the brain, body and different actors and artifacts embedded in a material-cultural ecology. So, rather than focusing on individual action, or invoking mental representations as a basis for cognition, we are inspired by Clark (2008) and Hutchins (2020), who see cognition as a process involving “the interaction of the consequences of past experience (for individual, group, and material world) with the affordances of the present. In this sense, culture is built into the distributed cognition perspective as at least a context for cognition” (377). In the same vein, Chemero (2011) proposes that all cognitive powers are seen as agent-environment interactions, where action is a result of a relational, structural coupling between an individual and its environment (Maturana and Varela, 1987). This combined perspective moves the object of investigation from the individual to the system, emphasizing the systemic features of human co-action.

By focusing on people and how they engage in whole-bodied activities that enable sense-making processes (Cowley et al., 2017; Trasmundi, 2020), we investigate *organizing* as a cognitive process emerging from the interactions among elements in a dynamic system, where organizations can be viewed as distributed networks of thoughts and actions, pointing at how we use each other and the environment as thinking resources (Gallagher, 2013). As recent work highlights (Slors, 2019), this view (i.e., Gallagher’s) aligns socially extended cognitive systems with the postulates of distributed cognition and allows us to define a more general framework for cognition. At the same time, together with Slors (2020) we acknowledge that both extended and distributed views of cognition do not place sufficient emphasis on the differences between social and artifact-based distributions. In our analysis, we focus specifically on two questions: What are the mechanisms involved in the emergence of organizational cognitive processes? And how can one capture aspects other than language and talk, when looking into organizational cognition? By taking a distributed and ecological perspective on organizations, we answer calls for a more “dynamic” and “complex” approach to the study of organizational phenomena (Langley and Tsoukas, 2010).

We conceive of *organizational cognition* in terms of *organizing*, whereby we contribute to research from a process-based philosophy on organizations (e.g., Langley and Tsoukas, 2010; Hernes, 2014), in particular we believe that our perspective can offer new insight into how people make sense and interact in organizational practice. In this, we find some overlaps with the sensemaking literature (e.g., Weick, 1995; Gioia, 2006), and the concept of collective mind (Weick and Roberts, 1993), and collective minding (Cooren, 2004). Despite a recent growth in the number of studies on *organizing* from a distributed and ecological perspective (Secchi and Adamsen, 2017; Cowley and Secchi, 2018), this paper offers a distinct contribution to the field by outlining an analytical framework as a way of demonstrating how a distributed and ecological approach to organizing can unveil aspects that are unlikely to appear by using more traditional research perspectives when analyzing organizing.

The paper is structured as follows: First it shortly introduces the most relevant theoretical elements of distributed cognition and provides the basic coordinates to the reader. Then, it presents the case, methods employed and analysis, and uses data to define the conceptual tools to answer the two questions above. Finally, it demonstrates potential operationalizations of the framework by feeding the conceptual structure onto an agent-based computational simulation model (Edmonds and Meyer, 2015), which is particularly suitable to support theoretical explorations in the social sciences (Secchi and Adamsen, 2017).

## Organizational Cognition: An Introduction

Mainstream cognitive science and philosophy of mind has traditionally taken biology as an individual phenomenon, while sociality is understood as something purely collective and public (Cuffari and Jensen, 2014). This “divide” is also apparent in cognition studies applied to organizations (Walsh, 1995). For instance, Hodgkinson and Healey (2008) review major developments within cognition in organizations and isolate five key theoretical perspectives pervading contemporary research, all of which seem to adopt a pre- or early-nineties approach to cognition (Secchi and Adamsen, 2017). They are: “(*a*) schema theory and related conceptions of mental representations […], (*b*) behavioral decision theory […], (*c*) attribution theory, (*d*) social identity theory, and (*e*) enactment and the related notion of sense-making” (Hodgkinson and Healey, 2008, 391). Seeing organizing as a sense-making activity has in particular been inspired by the framework provided by Weick (1979, 1995) and is perhaps the best-known process-oriented account in the field (Langley and Tsoukas, 2010), and it is probably one of the most advanced accounts of sensemaking processes in organizations. We therefore refer to the sensemaking framework when defining the contribution of a distributed and ecological framework to organizational cognition.

### Organizational Cognition Through Sensemaking

We find a rapidly growing interest in sensemaking within organization studies, (Cooren, 2004; Maitlis and Christianson, 2014; Brown et al., 2015; Sandberg and Tsoukas, 2015) making the sensemaking literature both very diverse and fragmented. In a review article of Weick’s sensemaking approach, Sandberg and Tsoukas (2015) point to the immanent ambiguity of the concept, which is partly due to lack of consensus as to whether sensemaking can be seen as individual-cognitive (mental maps), or specifically social and discursive (linguistic and communicative).

From a cognitive perspective, sensemaking has mostly been grounded in an individual internal process of the mind, relying on mental representations of reality, or as group-level consensus (Mohammed, 2001). As Weick and Bougon (1986, pp. 102–103) note, “organizations exist largely in the mind, and their existence takes the form of cognitive maps.” As noted by Cunliffe and Coupland (2012, 65) from this perspective sensemaking is mostly understood as a “rational, intellectual process represented through cognitive schemas and models.” Further, sensemaking is frequently seen as a retrospective process bound to the present where attention and meaning creation are directed backward, from a specific point in time (Weick et al., 2005; Langley and Tsoukas, 2010).

Other scholars see sensemaking as a social construction, giving the role of language, and communication a central position (Maitlis, 2005), relating sensemaking to conversational narrative processes (e.g., Boje, 1994; Brown, 2006). This is reflected in streams of studies that emphasize language, talk and communication as important aspects of sensemaking, (e.g., Cooren, 2004; Weick, 2012; Maitlis and Christianson, 2014). From this perspective, sensemaking is mostly conceived of as a social and discursive process, where the environmental context serves as a necessary background and input to the cognitive system.

Although extremely useful in many respects, these sensemaking perspectives significantly limit the explanatory and analytical powers of both communication and cognition. On the one hand, it reduces communication to talk and text, overplaying the role of conscious deliberate activities as opposed to more spontaneous semi-random or casual interactions, even if Weick attempted to correct this with the ideas of bricolage and improvisation; (Weick, 1993). On the other hand, it faces the risk of being an over-socialized (constructivist) account of human interactions in organizations (Cunliffe and Coupland, 2012; Maitlis and Christianson, 2014). In so doing, it does not fully consider, for example, the intrinsic meaning attached to artifacts and other external resources in the environment (Thompson and Stapleton, 2009).

However, more recent studies have started to focus on more embodied and embedded views on sensemaking (Cunliffe and Coupland, 2012) including sociomaterial, temporal and ecological aspects (e.g., Cunliffe et al., 2004; Feldman and Orlikowski, 2011; Whiteman and Cooper, 2011). These emerging developments within the sensemaking literature may help to overcome the above-mentioned limitations and help move the field of organizing forward.

But, in order to further advance our understanding of organizational cognition, we claim that it is necessary to go back to the roots of sensemaking – i.e., cognition (Weick, 2012; Maitlis and Christianson, 2014). In this, we refer to one of the most fruitful advancements in the study of cognition, one that is drawing on a distributed and ecological perspective (e.g., Hutchins, 1995; Hollan et al., 2000; Clark, 2008; Menary, 2010; Gibson, 2014; Cowley et al., 2017), which allows us to focus on the *interconnectedness* of agents, situations, relations and environment, thereby moving the object of analysis from the individual to the system.

### Assumptions of Distributed and Ecological Cognitive Processes

The perspective in this article on organizations sees cognition in terms of agent-environment as well as agent-agent dynamics unfolding over time rather than in terms of computation and mental representation (Chemero, 2011). This enables us to focus on the interconnectedness of cognitive resources (Clark and Chalmers, 1998) rather than on location – i.e., on whether they belong to one’s brain or to the tools one is using. This is also considered a “systemic” approach to cognition (Cowley et al., 2017).

Traditionally, there has been a sharp separation between what happens inside human beings – biological and cognitive processes, thoughts and emotions – and what happens between human beings – socially, linguistically and through communication. A distributed and ecological approach confronts the internal/external distinction and sees the study of cognition in contexts, which means that any individual is seen in direct relation with its environment. On the one hand, external and internal cognitive resources are intertwined in a way that it becomes very hard to draw a line between the two (Clark, 2008). On the other hand, there is an interplay among these resources (Clark and Chalmers, 1998) such that the ones affect the others in constant continuity.

The act of writing is an example of a distributed cognitive process, where one “externalizes” thinking using artifacts (e.g., computer, paper, pencil, the written words), and then re-internalizes the outcomes of this action, known as “re-projecting”; (Magnani, 2007) gaining a different perspective on the original in-brain activity. Simple tasks, such as compiling a shopping list of items, usually look different after one starts the externalization process and writes the items down. The action of writing down enables further thinking, and sometimes it allows to populate the list with items that one has not originally had in mind. In his approach to distributed cognition, Hutchins (1995) sees cognitive processes as distributed over space and time. This leads Hollan et al. (2000, 176) to specify that there are three “distributions:”

- Cognitive processes may be distributed across the members of a social group.
- Cognitive processes may involve coordination between internal and external (material or environmental) structure.
- Processes may be distributed through time in such a way that the products of earlier events can transform the nature of later events.

This approach emphasizes the interconnectedness of all parts of the cognitive process between internal, external, macro, meso, micro levels and the focus on social aspects (Hutchins, 1995; Neumann and Cowley, 2016; Secchi and Cowley, 2016; Cowley et al., 2017; Secchi and Adamsen, 2017). Defining organizing as a distributed and ecological process enables us to investigate it as a cognitive process emerging from the interactions among elements in a dynamic system (Hutchins, 2014). This has led others (e.g., Cowley et al., 2017) to hypothesize that the focus of the cognitive process may be on the system rather than on a particular way of distributing, manipulating, or defining cognitive resources. This “systemic view” emphasizes the ‘adaptive, coordinative and self-organizing nature of human interaction to the degree that human dialogue is viewed as a “functional whole.” Moreover, from an ecological perspective, a system that is open and complex presents emergent properties. It is therefore possible to frame a group of people engaged in interaction, such as face-to-face meetings as a bundle of “coupling mechanisms” (e.g., Clark, 2003; Chemero, 2011) with individuals as component subsystems that emerge from the relation between “subsystems of body, brain, and mind” (Cameron and Larsen-Freeman, 2007, 162).

## The Ø-Hop Case

The name Ø-Hop is completely fictional, and it is evocative of a summer activity in vogue in Denmark that consists in moving from one of the small islands surrounding the country to another. In fact, the letter “ø” is also a word meaning “island” in Danish, while “hop” is a short jump, like in English. We use this fictional expression to indicate how the individuals in the meeting switch more or less rapidly (or hop) from one *coupling mechanism* to another as the meeting unfolds. This mimics the frantic ø-Hop activity that some Danes entertain over the summer.

A significant part of organizational life is today spent in meetings, these are, perhaps, the typical representation of what happens in organizations; in fact, their dynamics, content, structure, socialization mechanisms, and rules of operations are a fair demonstration of the life of an organization. Meetings are a joint organizational practice with a particular focus on time, i.e., what happened in the past, what is happening in the present and, in particular, what will happen in the future, inviting a distributed cognitive perspective from the very outset. Accordingly, we see meetings as highly structured “dynamic” practices, distributed on and constrained by different timescales. They are the result of past decisions, and/or there may be references to prior decisions, and they are oriented to future meetings and possible future outcomes. Meetings may have participants with different sociocultural and professional backgrounds, they include invitations, an agenda, minutes taking, a specific physical setting (e.g., a meeting table, computers, pen and paper, etc.) and often a visual (power point) presentation — all acting as structuring devices in the making of the context. Moreover, meetings are “complex,” as multiple groups interact in flexible and unpredictable ways. This means that these physical objects as well as social constraints function as enabling conditions for the trajectory and outcome of the meeting. In the following we aim at identifying how people use each other and the environment to make sense, understand others and coordinate interaction.

### Data and Method

The data for this study is based on an ethnographic study in a Danish subsidiary of a large multinational company. The data selected for the present article come from a video recording of employees and management meeting, with nine people present, two females and seven males, and three different nationalities (Danish, German, Romanian), three managers (identified with M1, M2, and M3) and six employees (labeled E1–E6). The meeting was conducted in English.

**FIGURE 1:**
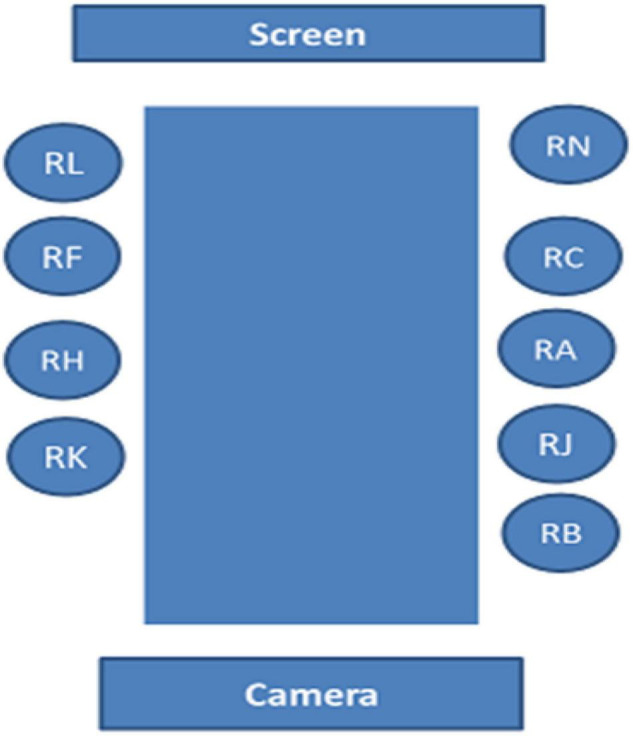
The room layout.

The recording is 2 h in total and was conducted by only one camera at the end of the table and was made by a graduate student working with one of the authors in a project on intercultural communication (see Figure 1). The name Ø-Hop is fictional to preserve anonymity and confidentiality.

The full video recording of the meeting was reviewed several times and transcribed for analytical purpose. The participants provided their written informed consent to participate in this study. In order to protect the identity of the organization and the participants in the meeting, all names have been anonymized.

The purpose of our analysis is to develop a framework that enables us to answer the following questions: What are the mechanisms involved in the emergence of organizational cognitive processes? And how can one capture aspects other than talk and text, when looking into organizational cognition?

When developing our conceptual framework, we draw on MultiModal Interaction Analysis (MMIA) (Goodwin, 2000). The rationale of MMIA is, in part, inspired by Conversation Analysis (CA) in the sense that it is designed to investigate the sequentiality of social action. This is a detailed study of how participants in conversation co-construct a social order by looking at how interactants, on a turn-by-turn basis, orient to and thereby exhibit their understanding of the state of talk (Hutchby and Wooffitt, 2008). This implies that the focus is not just on *what* people talk about, but on *how* they do it and what kind of patterns can be derived from this sequential ordering. This is often referred to as “next-turn-proof-procedure,” and it has created the basis for an evidence-based methodology that basically examines the social order of conversations, that is, how turns are sequentially structured in a turn taking-system (Sacks et al., 1974) such as, for instance, the sequential co-construction of question-answer patterns in various social settings. However, compared to CA, MMIA has an additional focus on human interaction as a whole-bodied activity embedded in a physical and social environment. At the heart of the method lies the assumption that the verbal and bodily non-verbal dimensions of language are equally important dimensions of the act of “doing language” (or “languaging”) with other people. Thus, MMIA combines attention to verbal actions with embodied actions, such as gesture, gaze, posture and facial expression (Goodwin, 2000; Cuffari and Jensen, 2014).

In order to support our theoretical argument, and in an attempt to demonstrate how the framework could be treated and, to some extent, generalized, we supplement the qualitative analysis with a computational agent-based simulation model (ABM) (Gilbert, 2008), using the software NetLogo 6.0.2 (Wilensky, 1999). The aim of the model is to demonstrate a potential application of our analytical framework.

### Contextual Information

The context of the meeting is the problem the company is facing as they are currently using a computer program called “SRM Tool,” but they will soon need to make a transition to another program called “Team Center.” However, their IT department in Germany does not support a transition from SRM Tool to Team Center, which is rather problematic. The management has therefore found a solution, which they will present at the meeting. The solution being a temporary transition to another system called “Doors.”

At the meeting, participants are supposed to discuss what changes are needed in order to solve the IT-problems and what requirements will be needed to make the transition from SRM Tool to Doors. The purpose of the meeting, however, is not quite clear to all participants, and the following two sequences (presented respectively in Boxes 1, 2) are taken from the beginning of the meeting at the time when they are trying to establish a “mutual purpose.” Thus, the agenda is built around (1) how to do the transition, and (2) when to do the transition. However, throughout the meeting there are recurrent patterns of interaction in which both (1) the agenda is questioned, i.e., whether they should rather discuss if they are to do such a transition, and, as a result of that, (2) the value of doing such a transition is – directly and indirectly – put to question. In other words, tensions arise when trying to identify and decide on a mutual purpose of the meeting. In particular, instead of resolving the problem by discussing how to do the transition, they rather find two different solutions to the emerging problem. Solution (a), as proposed by management, is a temporary transition to another system called “Doors,” involving two transitions (from SRM Tool to Doors and from Doors to Team Center). Solution (b), suggested by the employees, is to go directly to Team Center and skip the transition to Doors, which only involves one transition. The first solution appears to be easier on the technical side but heavy on regular employees, while the second is rather complicated on the technical side but easier for employees. This discrepancy gave rise to the discussion in the meeting.

**Box 1. Sequence 1.**

**Table:** 

L	P	Transcription	Time
1	M1:	…[…]-the intention of the meeting here is to I guess kind of brainstorm about ↓how can we move	S 2:01
2		These requirements – the requirements handling – into Doors [what]	E 2:14
3	M2:	[about] what is needed to move it (.) what we discovered in the summer when [*Name*] was on holiday for 5	S 2:14
4		Weeks ↓was that it felt a bit like nothing was working > the > process was not really known not really	
5 6		Implemented in the organization the tool was not working as it should people had a lot of questions and like that	E 2:36
7 8		*/M2 is making a movement with her hands while she talks, almost as if she is counting on her fingers as she lists the problems*	
9	M2:	So the situation was very bad for everybody and the frustration really was high by doing this meeting we	S 2:37
10		Want to have your input as users <what do you see is necessary in order to make the transition> there is no	
11		Doubt that we are going to make a transition that will come but we need what is needed in order to do this	
12		Transition and this feedback we need from you by doing this meeting we want to have your input as users	
13		<what do you see is necessary in order to make the transition> there is no doubt that we are going to make a	
14		Transition that will come but we need what is needed in order to do this transition and this feedback we need	
15		From you	E 3:01
16	E1:	A question – eh- what transition do you mean – the transition to Team center or – eh – ehm an earlier	S 3:02
17		Transition to – ehm – eh – like what you say – suggestion	E 3:14
18		*/Just as E1 poses his question E2 raises his hand and keeps it on and off for/Another employee [could not see who it is] says “ye-haaa” [approval]*	
19	M2:	I’m suggesting right now is to go to Doors	S 3:13 E 3:14
20	E1:	So you mean this transition SRM to Doors	S 3:14 E 3:18
21	M2:	Yes	3:18
22	E3:	S- sorry - so from SRM to Doors	S 3:19 E 3:21
23	M2:	°yeah°	3:21
24	E3:	Ahhhhhh this is absolutely new	S 3:22 E 3:24
25		*/E3 looks surprised at E1*	
26	E1:	It was in the invitation	S 3:24 E 3:25
27		*/E1 points to the screen*	
28	E3:	Yeah yeah yes – no no the invitation said future of SRM Tool	S 3:25 E 3:30
29		/E3 puts her glasses on the table	
30	E1:	[No it says our suggestion for]	S 3:30 E3:31
31	M2:	[(inaudible)] transfer to Doors before Christmas 2012	S 3:31 E 3:35
32	E2:	But then we will have two transitions [(inaudible)]	S 3:36 E 3:38
33	E3:	[just a] second I was thinking that [(inaudible)]	S 3:38 E 3:42
34	M2:	[(inaudible) the second] transition will be smooth	S 3:38
35		because this will not be our responsibility	E 3:43
36		/M3 walks to the whiteboard while E3 and M2 talk and begins to draw	
37	E2:	No transition is smooth	S 3:43 E 3:45
38		/some laughter	
39	M2:	No but if we continue with the srm tool we cannot expect the help from the it department in Karlsruhe	S 3:46
40		To make the migration (.)	E 3:55

*L, line; P, participant; Time sequence: S, start; E, end. M1, M2, and M3 are managers while E1–E6 are employees; italics indicates actions relevant to the discussion; text in [] identifies something that is said on top of another person’s talking.*

**Box 2. Sequence 2.**

**Table:** 

L	P	Transcription	Time
1	M3:	So what we know is that eventually we will end up in Team Center [eh]	S 4:07 E 4:13
2		*/M3 has drawn something on the board [see Figure 2] and points at the white board with his marker*	
3		*/M2 moves the chair making some noise*	
4		and it is also a fact that there will be a transition from Doors to Team Center it is also a fact that	S 4:19
5		There will be no transition made from SRM to Team Center from central pi (.) so now our	
6		Proposal is to transfer into Doors and then this is then automatically or by help by – at least then	
7		We are not alone	E 4:41
8		/M1 says something to M2 (inaudible)	
9	E3:	So that means it’s mandatory for all of us, or, anyway, highly recommended to use Doors, to install Doors	S 4:45
10		and start… (inaudible, voices overlap)	E 4:53
11		\E1 and E2 raise their hands	
12	E1:	E2 was first	S 4:57 E 4:58
13	E2:	Why not a transition directly for Team Center on our own (.) I think the Team Center modules are	S 4:58
14		Available (inaudible) at least what I hear is that they are available [(inaudible)]	E 5:13
15	M2:	[it’s a suggestion]	S 5:09 E 5:10
16	M1:	We had that meeting where we were demonstrated this feature and it didn’t have version control on	S 5:13
17		The requirements I would say that it is not finished yet	E 5:23
18	E2:	I heard different	S 5:25 E 5:26
19	M1:	Were you in the meeting as well	S 5:26 E 5:27
20	E2:	I don’t know what meeting you refer to (inaudible) they are using the: (.) modules I believe	S 5:27 E 5:38
21	M3:	So what you mean E2 is to go this way	S 5:39 E 5:45
22		/M3 walks to the white board again and draws an arrow	
23	E2:	That is not in the current version of Team Center we have…	S 5:45
24		it’s in a future version	E 5:47
25		/some brief inaudible exchanges while E6 moves the chair (making some noise)	
26	E6:	But I guess we did not upgrade to the latest version of Team Center with this upgrade…	S 5:55
27		/E6 pauses and looks in the direction of M1 (where M2 is also standing behind and next to the board)	
28		we just did…	E 6:00
29	E2:	[I don’t think so]	S 6:00
30		But Team Center is not just what we know, there are there are lots of other models (inaudible)	
31		and I know [Name] in marketing is… have asked if we are allowed to…because they have also	
32		Postponed managing what you are saying, but he would like to run a trial and the (inaudible) would be great	
33		If we could (inaudible)	E 6:21
34	M2:	The intention is not to discuss solutions now	S 6:22 E 6:24
35	E2:	[Ok]	6:24
36	M2:	The intention is to clarify our mutual purpose to agree upon what it is we are doing	S 6:24
37		and then come up with [h] suggestions; your suggestion is good and I invite you to repeat it when I start	
38		Typing the minutes so that we get this idea as well	E 6:39
39	E2:	But I need to know if it has been decided to go to Doors, is this meeting to discuss if we should go to Doors?	S 6:40 E 6:45
40	M2:	No, the decision is we should go to Doors	S 6:45 E 6:47
41	E2:	/looking at the email	
42		It just says it is a suggestion and I also thought the meeting was to evaluate these (inaudible)	S 6:47
43		So, the meeting is just to discuss what is needed to go to Doors	E 6:57
44	M2:	[Yes]	6:57
45		\some nodding	

*L, line; P, participant; Time sequence: S, start; E, end; M1, M2, and M3 are managers while E1–E6 are employees; italics indicates actions relevant to the discussion; text in [] identifies something that is said on top of another person’s talking.*

We selected these two sequences because (a) they represent a situation that has happened at least once (most likely several times) to anyone taking part to a meeting, (b) the controversy over such a simple point (an item in the agenda) makes it easier to detect cognitive processes.

Further, the interactions are not always clearly detectable from audio only, it is therefore necessary to include video in order fully to understand and make the role of resources and cognitive mechanisms more apparent.

In the subparagraph below, we offer a brief description of two sequences extrapolated from the meeting, in an attempt to highlight aspects that will be relevant to build the conceptual framework as presented in the next section.

### Data Description

Box 1 presents a detailed transcription of the first sequence, covering the initial part of the meeting (1 min and 54 s), where the interactions are particularly revealing of the starting conditions in which members find themselves.

**FIGURE 2:**
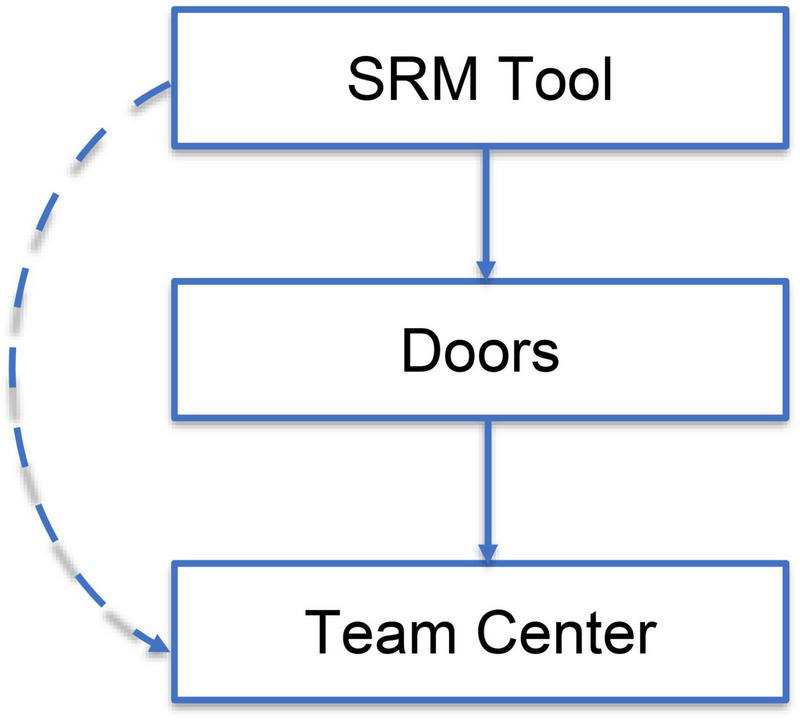
Replica of the picture on the board.

At one point, M3 goes to the white board and makes a drawing in order to explain the purpose of the meeting. He then draws arrows between the boxes to illustrate how the transition will be carried out (reported in Figure 2).

If we look at the above sequence, something noticeable happens from line *1–15*: Two representatives from management have been accounting for their reasons to call for this meeting, and they have presented their solution – move the system to Doors – to an upcoming problem in relation to a shift to a new software. The decision is based on several problems which the management “discovered in the summer” (line *3*). In line *16*, however, one of the employees (E1) asks the question: “a question – eh – what transition do you mean – the transition to Team Center or – eh – ehm an earlier transition.” Evidently, it is not clear to him what kind of transition they are in fact talking about, and this is not just the case for E1. Upon receiving the answer in line *19*, that the transition in question is a transition to Doors, another employee (E3) reacts promptly. From line *20–29* a series of adjacency-pairs (question-answer) between E3, M2 and E1 show that not only had E3 also been oblivious about the nature of the transition, she also resents not having been sufficiently informed before the meeting. By looking more carefully at the video recording, we see that, at the beginning, E3 is leaning back in her seat looking at M2, who is doing the talking. However, a shift occurs in line *11* just after M2 has uttered “there is no doubt that we are going to make a transition, that will come” – E3 suddenly leans forward and carefully studies the screen that displays the email invitation. Apparently, the information that “a transition will come” prompts her to scrutinize the actual text of the email invitation, again suggesting that the information about a transition is new to her. This is confirmed in line *24* in which E3, upon receiving an affirmative response to her question about the transition, raises her voice and clearly displays a token of surprise (“Ahhhhhh”), explicitly verbalizing her surprise: “this is absolutely new.” The rest of the sequence is a discussion between two of the employees, E1 and E3, as well as between employees and management, E2 and M2, whether the information about the transition (from SRM to Doors) is indeed “NEW” information, or if it had been communicated in the email invitation. A discussion follows about whether such a transition can run smoothly or not. In this argument, M2 invokes the department in Karlsruhe as a powerful absent “other” as an outside third party (Linell, 2009) influencing the present conversation in the meeting.

**FIGURE 3:**
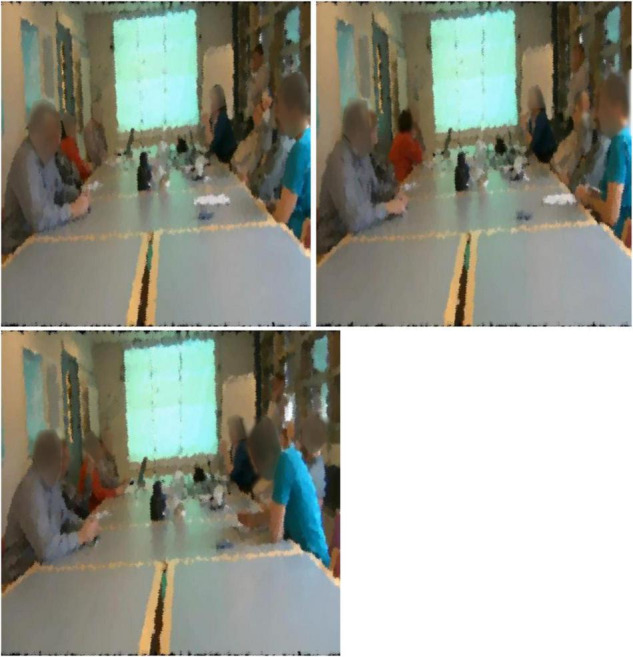
Three sequences from the video recording.

The second sequence is about 3 min later into the recording and lasts 2 min and 50 s (see Box 2). It is still a part of the official meeting agenda – the temporary transition from SRM to Doors – and it is still under discussion and still being questioned. The participants discuss the possibility of an alternative solution, i.e., to go directly to Team Center, instead of going to Doors first, and then move to Team Center, i.e., to have one transition instead of two. To give a livelier picture of the situation, Figure 3 presents three snapshots taken in different moments of the meeting.

M3 starts this part of the meeting by stating that there are indisputable facts, and these are the end point of the transition (“eventually we will end up in Team Centre”) and that this cannot go directly from the current software (i.e., SRM) to Team Centre (lines *4–7*). In line *13* (Box 2), E2 introduces a new solution, when he argues that it would make more sense to make a transition “directly for Team Center on our own.” In answering his question in lines *16–17*, M1 refers to a discussion and a conclusion on that discussion that took place in a previous meeting during which the “version control” of Team Center was addressed. Based on that, M1 concludes: “I would say that it is not finished yet.” However, directly questioning M1’s interpretation of this discussion, E2 insists on a different version of that discussion by simply saying: “I heard different” (line *18*). This way of blatantly questioning the manager’s version in front of the other managers and employees can potentially be construed as face threatening, in particular if it might appear that M1 does not remember correctly or chooses to make his own interpretation. However, instead of going into the discussion about what was said and concluded, M1 decides on another strategy, namely that of questioning whether E2 had actually been to the meeting: “were you in the meeting as well” (line *19*). In that way, he manages to change focus away from his own conclusions about the meeting to a question about E2’s presence, i.e., the right of E2 to have an opinion about this or not.

Again, this has clear normative implications, as can be seen in this example. If you are not part of, or aware of, the chain of meetings it can be difficult to “jump in” and have a say, as your opinion, and ability to navigate in the here-and-now will always be constrained, and judged, by your history in, and knowledge about, the organizational context in general, and your activities, or lack of activities, in particular. In this case, E2 tries to avoid being held accountable for his (non-)presence at the earlier meeting by saying (in line *20*) that he does not know “what meeting you refer to.” Still, the question about M1’s interpretation of what was decided at the previous meeting, is not pursued in the following discussions. The fact that the manager sheds doubt about E2’s presence at that meeting seems to prevent any further discussion on this. The sequence ends with M3 walking to the white board again drawing an arrow showing the trajectory of possible “transitions,” thereby using the whiteboard to demonstrate the two possible solutions to the problem.

In the final part of Sequence 2, E2 and E6 discuss briefly (lines *23–33*) about upgraded versions of the software Team Centre or lack thereof, that would make it possible for them to have a direct migration. At one point, M2 breaks the discussion to state that this discussion is irrelevant because “the intention [of the meeting] is to clarify our mutual purpose to agree upon what it is we are doing” (line *36*) and to offer suggestions on how to do it. Again, E2 wants to be absolutely sure that this is the point and M2 confirms that “the decision is we should go to Doors” (line *40*). At that point, E2 repeats “so, the meeting is just to discuss what is needed to go to Doors” (line *43*). Some agreement signs come from M2 and from around the room. Finally, one of the points in the meeting’s agenda seem to have settled.

## Cognitive Mechanisms in the Ø-Hop Case

As seen in the brief introduction above, distributed and ecological cognition places a very strong role in the interactivity, (interplay); (Hutchins, 1995; Clark and Chalmers, 1998; Steffensen, 2013) between internal and external resources. Hence, any cognitive mechanism should be based on a classification of these resources. By using the Ø-Hop Case, we now attempt to build a conceptual framework that is relevant to organization research. As we move on in the description, a brief summary of core “mechanisms,” “resources,” and “processes” can be found in Table 1.

**TABLE 1:** Cognitive mechanisms, resources, and processes.

Mechanisms	Resources	Processes			Relevant DEC literature
		*Coupling*	*De-coupling*	*Un-coupling*	
Socio-Material (CM1)	(*i*) People Case-related examples	Observable active participation in the interaction	The individual stops interacting with external social resources	There is no clear sign (emotional, verbal, behavioral) of activity or interaction	
		*Example: M2 and E1 agree on the meaning of the transition (Sequence 1, line 21)*	*Example: E3 was under the impression that E1 was in line with her, but E1 points out that information was in the email (Sequence 1, lines 25–26)*	*Example: E4 and E5 are in the same room as the others but they do not talk or even look at each other*	***Relevant DC literature:*** *Hutchins, 1995; Hollan et al., 2000; Magnani, 2007; Secchi, 2011*
	(*ii*) Artifacts Case-related examples	There is a clear way in which one uses or exploits an artifact available to either reason or to give a sign to others	The artifact is not used to establish any sort of relation but it is just a means to an end	There is no distinguishable sign that one is using an artifact for any constructive purpose	
		*Example: M3 uses a marker on the whiteboard, allowing himself to explain again (differently) a concept previously expressed only verbally (Sequence 1, line 36)*	*Example: E2 uses M2’s statements to de-couple himself from the text of the email (Sequence 2, lines 41-43)*	*Example: fiddling with a pen on a piece of paper, drawing lines at random (maybe a sign of boredom?) instead of taking notes*	***Relevant DC literature:*** *Hutchins, 1995; Clark, 2003; Bardone, 2011; Cowley and Vallée-Tourangeau, 2013*
Conceptual (CM2)	(i) Idea of the group/organization Case-related examples	Individuals may engage with others more easily if they have a positive attitude toward the group/organization — this can be exemplified by commitment, identity and identification, justice, and other beliefs one may have of the place of work **N.B.**: this particular feature of CM2 can be referred to more specific relationships between, for example, two co-workers	One’s positive representation of the group/organization may deteriorate over time, provoking a decreased engagement	An individual may feel as an outsider and never really get in tune with a positive idealized understanding of what the group/organization is there for	
		*Example: M2 has a clear idea of what the meeting is there for and what her role is (Sequence 1, lines 3–6)*	*Example: E6 brings in new information, with the idea to re-route the discussion on more technical grounds (Sequence 2, line 26)*	*Example: When E3 presents her disagreement, she seems to signal she is an uninformed outsider (Sequence 1, line 24)*	***Relevant DC literature:*** *Michel, 2007; Secchi and Bardone, 2013*
	(*ii*) Topic understanding Case-related examples	Mutual understanding ought to be based on the perception that there is some shared understanding of the topic under discussion	Sometimes people diverge in their understanding and this causes a diversity of meanings attached to the topic under discussion	Complete lack of understanding of the topic under discussion	
		*Example: Toward the end, E2 and M2 agree (with many others) that the meeting is to discuss how transition will happen (Sequence 2, lines 44-45)*	*Example: E2 attempts to detach himself from the two-step transition by proposing a one-step transition (Sequence 2, lines 13-14)*	*Example: We do not know about E4 and E5; for the purpose of the analysis, they seem uncoupled with the understanding of the topic as discussed.*	***Relevant DC literature:*** *Magnani, 2007; Michel, 2007; Secchi and Gullekson, 2016*
Conceptual (CM2)	(*iii*) Meaning of procedures in place Case-related examples	Organizational procedures (formal and informal) are a good conceptual anchor for those who seek certainty	Some procedures (formal and informal) may be interpreted differently by some, hence causing temporary or permanent confusion	There are no expectations of how procedures would unfold in a group/organization	
		*Example: M1 presents the idea that procedures are up for discussion when he states that they are there to “I guess kind of brainstorm” about the transition (Sequence 1, line 1)*	*Example: M2, with a more specific explanation of how the meeting would be handled de-couples from what indicated by M1 (Sequence 1, lines 3–6)*	*Example: M1 and M2 ideas of how to proceed seem unpaired and opposite (Sequence 1) [although M1’s silence may look like an attempt to couple with the idea]*	***Relevant DC literature:*** *Hutchins, 1995, 2013*
	(*iv*) Perceptions of time Case-related examples	Whenever two or more individuals interact, they lean on past interactions and, at the same time, keep thinking of future consequences of the current interaction	The interpretation that two (or more) individuals have of past of future events may diverge, causing a temporary or permanent disengagement from the interaction	The interpretation of that two or more individuals have on past or future events may be completely different	
		*Example: M1 explicitly refers to a previous meeting (Sequence 2, line 16)*	*Example: Perhaps in an attempt to swing the discussion, E2 states that the transition is time-sensitive referring to the marketing department (Sequence 2, lines 30–33)*	*Example: M1 and E2 seem to have a completely different memory of another meeting; they do not agree on when or what (Sequence 2, lines 16-20)*	***Relevant DC literature:*** *Cowley and Vallée-Tourangeau, 2013; Cowley and Steffensen, 2015; Neumann and Cowley, 2016*

### Socio-Material Cognitive Mechanisms

Meetings are usually distributed among different artifacts such as invitations, minutes, and also socio-physical “resources” such as, for instance, computers, white boards, projected images. This comes up very clearly from the Ø-Hop Case as well. For example, the email is projected on a large screen by the wall as Sequences 1 and 2 take place and sometimes the conversation explicitly refers to it (Sequence 1, line *27*; Sequence 2, line *41*). There is a board with mobile white paper that M3 uses in combination with a marker (Sequence 2, line *2*) to draw a “transition model” (Sequence 1, line *36* and Figure 2). The minutes are also mentioned as M2 is willing to take E2’s suggestion on board (Sequence 2, lines *36–38*). One’s own body is also part of a distribution in a cognitive system, so that raising a hand (or keeping one’s arm up) serves as a signal to others and as a reminder to oneself of the function one is about to perform (Sequence 1, line *18*; Sequence 2, line *11*). This also serves as a social hook, as a basis to signal that communication is about to start, that someone is about to share something with the rest of the group. Most of these examples have at least a double meaning. On the one hand, they represent an anchor to the material or natural artifact they explicitly refer to (we call them “material resources” or “artifacts” (resource *ii* under CM1 in Table 1 – CM1, defined below). On the other hand, they are intrinsically social in that they are either means of connection to others or are (more literally) others (called “social resources” or, perhaps more simply, “people”; resource *i* under CM1 in Table 1). For this reason, we propose to classify these as “socio-material resources” that work as cognitive enabling (or disabling) tools in what can be described as cognitive mechanism one (CM1) and are clearly related to the strategic trajectory of the events.

In this case, that of ‘‘socio-material coupling mechanisms’’ (CM1), individuals attempt to ‘‘adjust’’ to one another and to the group^1^ (Hutchins, 1995, 2014). This means that there is no simple “coupling” (in its literal sense of only two entities affecting each other) but a bundle of couplings, which include mixed series of cognitive processes. This points to the dynamics in a group, assuming there are multiple interactions with members and with the group, all potentially occurring at the same time (the literature on small group dynamics is particularly revealing; e.g., Levine and Moreland, 2012). This perspective is also in line with the earlier literature on distributed cognition that sees cognitive processes as triggered by artifacts, (e.g., Hutchins, 1995; Clark, 2003; Magnani, 2007) seeing material artifacts and environmental structures as resources for interaction. There is also some overlap with the sensemaking literature, (e.g., Weick, 1993; Weick and Roberts, 1993; Gioia, 2006) in that these socio-material couplings are usually triggered by action in a given situation. The concept of “coupled systems” has been used among distributed cognitive (scholars) for quite some time, but never applied to organizational contexts (Clark and Chalmers, 1998; Menary, 2010).

### Conceptual Cognitive Mechanisms

By looking into the Ø-Hop Case, one soon realizes that there are other types of mechanisms that are not necessarily tied to material or social cognitive resources. It is the case when anchoring is made to an idea, thought, or to something abstract that is immaterial. In one instance, for example, one of the managers (M2) appeals to the “identity” of the group and calls for a final agreement on the meaning of the item in the agenda that was discussed for almost 7 min (Sequence 2, lines *36–38*, and again in line *44*). In many other instances, instead, participants struggle to understand what the purpose of the meeting is; for example, E1 in Sequence 1, lines *16–17*, E3 in Sequence 1, line *24*, as well as E2 in Sequence 2, line *39*. In these occurrences, comprehension is at stake and we can clearly observe participants as they try to grasp the meaning of what is the matter of the topic they are discussing. In the first case, cognition is anchored to the *idea of the group* (or the organization, depending on the situation; see Table 1, resource *i* under CM2—see below for a definition of CM2) while it leans on *topic understanding* (Table 1, resource *ii* under CM2) in the second case.

The Ø-Hop Case also presents other types of abstract anchoring. For example, how interaction materializes is something that may or may not happen in the making, i.e., as the meeting (or, more generally, the interaction) progresses. At the very beginning, there seems to be uncertainty over *how* the discussion is going to take place. In fact, M1 presents the idea that procedures are up for discussion when he states that they are there to “I guess kind of brainstorm” about the transition (Sequence 1, line *1*). That is immediately rephrased by M2, with a – clearer, at that point, she probably hoped – more specific explanation of how the meeting would be handled (Sequence 1, lines *3–6*). In several other parts of the meeting, a structured process emerges from more confusing phases, where voices overlap and local couplings (i.e., not at the general group level but at the level of two or three persons interacting) happen. This is, for example, apparent when E3 looks surprised at E1 (Sequence 1, line *25*), or when M2 and E3 discuss as M1 attempts to explain what he means by moving toward the board (Sequence 1, line *36*). Or, again, when M1 chats briefly with M3 (Sequence 2, line *8*), appearing detached from the meeting for a moment. Table 1 indicates these as “procedural meanings” and labels it resource *iii* (under CM2).

Finally, and perhaps, most importantly, there are several occasions in which participants refer to “time.” This is not just the objective measurement of time but includes its perception as it is experienced by each participant (Table 1, resource *iv* under CM2). In the Ø-Hop Case, this becomes apparent many times. M1 refers to a previous meeting (Sequence 2, line *16*) as well as E2 (Sequence 2, line *18*), as if the current timescale is somehow affected by either past timescales or a meta-timescale that super-orders meetings on that topic. E2 comes back to the point that the transition is time-sensitive when he refers to something attempted by the marketing department (Sequence 2, lines *30–33*). M2 is, instead, very much concerned with the here-and-now and calls participants back on what she believes is most relevant in two different occasions, respectively at the beginning, in an episode with M1 (Sequence 1, lines *3–6*) and at the end with E2 and everyone else (Sequence 2, lines *36–38*).

In all these four circumstances, we are in front of “conceptual coupling mechanisms” (CM2), where group members try — both individually and as a group — to get attuned with the activity they are engaged in. We are not claiming that these processes are all necessarily conscious, (Levinthal and Rerup, 2006) actually, we are not making any claim on this particular aspect. In fact, most of the elements that enable these processes are embedded in the way group members approach and deal with ongoing activities.

While sometimes distributed and ecological processes need actual artifacts to be triggered (see above), some other times they need this type of “abstract” anchoring. Given the blurred and uncertain nature of abstractions, similar anchors may have a very diverse impact on different group members. Emotions such as fear may illustrate the case of an abstract anchoring mechanism on cognition. In their review study on entrepreneurs, Cacciotti and Hayton (2015) found that fear of failure can be both beneficial and detrimental for entrepreneurs who experience it, because it anchors current cognition to past experiences. In this same stream of literature, de Mol et al. (2015) define team cognition as an emerging property of the system, originating from individual cognitions. This means that whatever is shared at that level serves, again, as abstract anchoring for all team members. This aspect is probably more in line with recent developments in the literature on distributed cognition and cultural ecosystems, where the emphasis has been on cultural niches (e.g., Hutchins, 2014; Secchi and Cowley, 2016).

## Cognitive Processes in the Ø-Hop Case

These multiple coupling activities usually go on simultaneously and can be seen as attempts to create meaningful interactions, although not necessarily leading toward a shared view of what is discussed. In fact, together with these mechanisms one should look at the way in which cognizing emerges.

There are at least three possibilities here, as one may observe (a) coupling, (b) de-coupling, or (c) un-coupling processes. Specific examples on each of the six resources as they relate to these processes and the Ø-Hop Case can be found in Table 1. Here, we limit our attention to one example per process.

The first – i.e., “coupling” – is what has been defined so far and indicates how group members’ cognitions align to socio-material and conceptual anchors or resources, with other members and with the group as a whole. The case study presents several examples of this process. Perhaps, the most explicit can be easily detected by positive reinforcements of agreement (typically a “yes,” a nod, a smile, or other expressions of the same type). This happens toward the end of the meeting very explicitly when M2 gives confirmation about the purpose of the meeting (Sequence 2, line *44*). By that point in time, most participants seem to agree on this one item in the agenda they have been discussing (Sequence 2, line 45).

The second process refers to the disengagement of one’s cognition from the group, others, and/or from artifacts. Hence, it refers to something that was coupled before and is no more. This “de-coupling” is also apparent in the case study, when E3 brings herself off the current discussion by asserting the topic is a complete stranger to her (Sequence 1, line *24*). In that way, she takes herself out of coupling with the idea (CM2, resource *ii*) and, perhaps, with the understanding of what the group has been gathered there for (CM2, resource *i*).

The third alternative refers to the impossibility, for an individual, to establish a connection to either a material artifact, a particular group member, or to an abstract concept that is used as an anchor for the group to function. In this case, neither socio-material (CM1) nor conceptual (CM2) mechanisms work, and there is no interaction. This aspect can be referred to E4 or E5 who never say anything, nor seem interested in the discussion (from watching the video, at least). Of course, it is very hard to make this statement by observations only, but an “un-coupled resource” process on, say, one socio-material resource (CM1) may well be accompanied by “couplings to” some other resources from CM1 or CM2.

There is a tradition for cognition research to study mostly what occurs under letter (a) “coupling” processes (Menary, 2010), i.e., to study positive cognitive occurrences (Steffensen, 2013), while organizational cognition has traditionally taken (c) “un-coupled” processes, by studying interaction among separate, independent resources and individuals (Secchi and Adamsen, 2017). We claim, however, that all three are equally important in understanding *when* and *how* cognitive group dynamics works.

By no means do we maintain that one of these processes is positive while the others are negative, rather we encounter a series of ups and downs among members of a group at a meeting, for example. Failure to reach a common understanding may well be derived from too many un-coupled resources or sudden coupling and de-coupling processes, where individuals do not engage with either CM1, CM2 or both, or they stop doing so. Moreover, we maintain that any organizing activity is made of multiple processes, where a complex mix of coupling, de-coupling, and un-coupled processes attach themselves to the many combinations of resources.

What is relevant for our argument here is that, when organizational activities are ongoing individuals do not function in isolation. If we take the example of working meetings, there is an attempt that individuals (and groups) make to establish coupling mechanisms to connect with the activity “in the making” (or “through doing,” see Magnani, 2007). These mechanisms may be established through coupling, cease to exist via a de-coupling process, coupling in smaller sub-groups, or cannot be established at all (i.e., they are un-coupled).

A deeper understanding of these processes also implies that there are many aspects that affect organizational cognitive processes that are not explicit and conscious actions — we are referring to written and spoken words. In fact, everything related to the bodily expressions, including but not limited to posture, mood, feeling, eye movement, arms and legs movement are relevant to assess the direction in which the organizational cognitive process goes. Interpretation of both explicit and implicit actions needs to be considered very carefully, since there may not be alignment between the two. In the following section we have attempted to render the processes described here such that they can be considered in their general applicability.

## A Computational Model of Cognitive Dynamics

**FIGURE 4:**
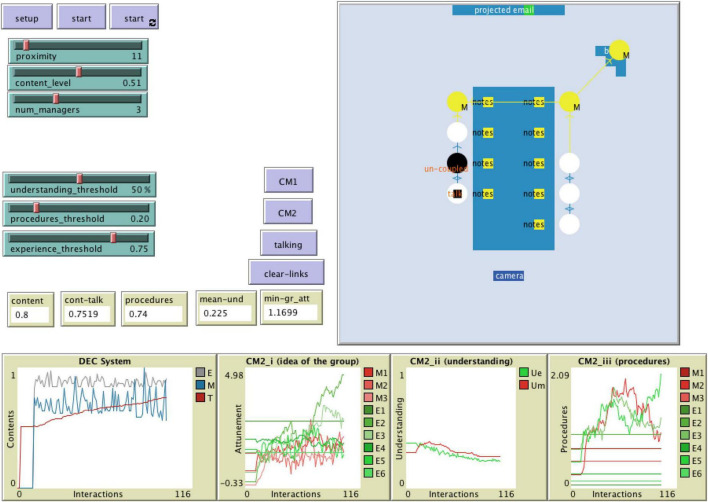
NetLogo interface: the base example. The interface presents the end result of a model where couplings from the various cognitive mechanisms (CM1 and CM2) are set to replicate the outcome of the meeting—i.e. agreement on one item of the agenda—, after 100 interactions.

The case study above highlights a few elements that can be considered as the basis for framing how organizational cognition actually happens. In the following, we present a computational ABM (Gilbert, 2008) developed using the software NetLogo 6.0.2 (Wilensky, 1999). Figure 4 shows the interface of our model. A version of this model, with Supplementary Materials and data is available on the open access platform OpenABM. ABM simulation is an advanced technique that has proven to work well with organizational behavior research (Secchi, 2015; Secchi and Neumann, 2016), especially with cognition-related matters (Conte et al., 1997; Cowley and Secchi, 2018). This model serves two purposes. One is to standardize the analysis of the qualitative empirical case and demonstrate that this approach can be applied to a variety of settings. The other is to illustrate a possibility, namely, that of studying complex organizational cognitive dynamics (such as those happening in meeting) through agent-based computational simulation (Secchi, 2021). For these reasons, we do not present a full set of results for this model, but only the specific configuration of parameters that fits the empirical case analyzed above as a way to validate this ABM (Boero and Squazzoni, 2005). The description below follows aspects of the ODD protocol for ABM (Grimm et al., 2020) while more detailed information is available in the Supplementary Materials File.

### Model Components

The aim of the model is that of demonstrating a possible application of the framework, with the simulation model being the bridge to either further virtual or empirical explorations. In the following, we quickly describe the components of the ABM and indicate which configurations of parameters replicate observations of the meeting above. The basic parameters and their values are described in Table 2 where column two indicates the possible range of values each parameter can take and column three shows the values that replicate the results (see below).

**TABLE 2:** Parameters and notations.

Parameters	Value range	Set values	Note
*hierarchy*	≈*N*(0, [0.1,1])	≈*N*(0,1)	Attitude toward the role of other persons; distributed normally at random among agents (CM1), with mean 0 and standard deviation 1. Low values are more likely to activate use of material resources while higher values activate other agents as cognitive resources.
*group_attunement*	≈*N*(1,[0.1,1])	≈*N*(1,1)	Attitude toward the group’s identity—CM2(i); distributed normally at random among agents, with mean 1 and standard deviation 1.
*understanding*	*random* [α]	α = 1	Own and shared understanding of the topic—CM2(ii); distributed randomly with numbers between zero and α (1 in this case)
*procedures*	*random* [β]	β = 1	Own and shared understanding of the procedures in place at the meeting—CM2(iii); distributed randomly with numbers between zero and β (1 in this case)
*acquaint*	≈*N*(0,[0.5,1])	≈*N*(0,1)	An agent’s knowledge of other agents from before this particular meeting—one dimension of long-term timescales, CM2(iv); distributed normally at random with mean 0 and st. dev. 1.
*experience*	*random* [γ]	γ = 1	An agent’s experience in the role it covers—one dimension of long-term timescales, CM2(iv); distributed randomly with numbers between zero and γ (1 in this case).
*content*	[0, 1]	*DoI*	The object that agents attempt to interpret with access to cognitive resources in the model. This is a function of the number of cognitive resources used for each agent; for the agent “talk” it is specified by the slider “content_level.”
*content_level*	[0, 1]	0.51	The parameter is attached to the “talk” and identifies the minimal content from which the conversation starts; it is modified by interactions as the simulation starts.
*und_threshold*	[0%, 100%]	50 %	The minimum level below which resources related to CM2(ii) are not activated.
*proc_threshold*	[0, 1]	0.20	The minimum level below which resources related to CM2(iii) are not activated.
*exp_threshold*	[0, 1]	0.75	The minimum level below which resources related to CM2(iv) are not activated.
*proximity*	[10, 20]	11	This is the scope of interaction. Lower values indicate that each agent only interacts with those next to it while the highest range value puts each agent in contact with everyone else in the simulation.

*DoI, depends on interactions, the function varies and it is specified in the additional materials online (OpenABM).*

The ABM features two different types of agents. Some agents are made to simulate the presence of people in the room, and they can be either managers or employees. Another agent-type simulates the talk. This latter agent is initially associated with the agent-person who is programmed to start the conversation at the meeting. Once created, this agent-talk moves from agent-person to agent-person depending on whether they are speaking or not. The agent-talk is characterized by a specific content, initially specified by parameter “content level” (Table 2).

There are nine simulated agent-persons in the model of which some can be set to be managers (labeled M; Figure 4) while the remaining are employees. To describe the coupling mechanisms, each agent is initially attributed attitudes toward the other agents on a random normal distribution (to represent CM1[i]; see Table 2 for details) and could interact with material resources in range of one’s attention (CM1[ii]). These are controlled by parameter “material” (see Table 2 for details). Also, each agent has an idea of the group (CM2[i], parameter “group attunement”) and has an understanding of the topic under discussion (CM2[ii], parameter “understanding”) as well as procedures in place (CM2[iii], parameter “procedures”). Finally, each agent has some experience and acquaintance with other agents, to represent a time-sensitive longer timescale aspect (CM2[iv], parameters “acquaint” and “experience”).

Material resources include a rectangular table in the middle of the simulated room, with a screen on the one side and a camera on the other. A board is positioned on the side, in between the table and the screen, and each agent has notebooks in front of them (Figure 4).

### Model Procedures

Some of the parameters of the model can be modified through the sliders on the interface (Figure 4), some others require intervening on the code. The ABM is designed to let agents interact in order to modify, adapt, or temporarily eliminate parts of their contribution to the cognitive processes. Most parameters described in Table 2 vary depending on the interactions that form, develop, or cease to exist during the simulation. These interactions are visually recognizable by color (lime and yellow) and by the oriented links between cognitive resources. A link identifies an active cognitive process between an agent and any of the other resources – these links are activated depending on proximity and combinations of the agent’s characteristics. These characteristics serve as thresholds that allow for the various cognitive mechanisms (CMs) to materialize. The interactions that stem from agent activities are, to use the words of our framework, the *coupling* processes. An interaction that ceases to exist witnesses an *un-coupling* process. Agents that are not engaged with a particular resource become black and are *de-coupled* (there is one in Figure 4).

The simulation also models talking. For simplicity purposes, this ABM does not allow multiple talks at the same time, but one squared agent with the orange label “talk” (Figure 4) appears and jumps from one agent to the other, depending on whether particular cognitive processes make it compelling for the agent to “say” something. The other agents may or may not pay attention to what this agent is “saying” (black links) and could reply. These processes obviously help to describe and set the content of the meeting for the agents. The plot at the bottom left corner of Figure 4 shows the overall interpretation that managers (dark blue line) and employees (gray line) have of the discussions during the meeting. The red line is the content that is shaped by the talk. So, in general, one may see that, on average, agents oscillate between different levels of their interpretations and the talk tends to position itself in between the two average interpretations of managers and employees (at least in this particular run of the simulation shown in Figure 4).

To be more precise in our description of these procedures, we can exemplify how CM[ii] works. The following pseudo-code offers a concise explanation:

FOR agent-employee connected to agent-talk
IF agent-employee connected to agent-manager
THEN set *understanding* ± gap
OTHERWISE set *understanding* + gap

Where the “gap” is the distance between the understanding that the two connected agents have of the content of the talk. This makes every interaction grounded on the initial understanding of each agent who is listening to the talk. Understanding is likely to fluctuate when the connection is with an agent-manager while the gap reduces when the connection is with another agent-employee. This is due to what observed in the empirical data, where employees would seek “alliances” with other employees more often than with managers. While the pseudo-code above works for agent-employees, there is a similar one working for managers. The specific value for the changes in understanding levels are different in the sense that they move more slowly — we assume management has stiffer positions as per the empirical data from the study above.

### Validation

Figure 4 shows the result of one run of the simulation after 100 interactions. The idea is that every step in the simulation represents one or more lines in the empirical analysis. Obviously, the analytical lines are not going to map exactly on the steps of the simulation but given certain configuration of parameters, the ABM replicates the outcome of the Ø-Hop case. In other words, after 100 opportunities for interaction, cognitive processes of the agents may converge on a particular content; as it happened in the case. This is a validation process in which the model has been made to systematically take different parameter values until the outcome converges with that of the case. The selection of values that allows for this convergence to happen is reported in Table 2.

In this simulation we are not aiming at explicitly modeling distributed and ecological cognitive processes, but to instruct agents to act in a way such that those dynamics may emerge under certain conditions.

### Possible Applications of the Model

There are many uses of a model such as the one we have presented here. By manipulating the parameters’ values, one may, for example, study how quickly agents reach agreement over interpretation of content, or what are the conditions for not reaching such an agreement. One may also study how many unengaged agents (reducing the impact of the mechanisms) it may take to sabotage the meeting. Or whether more interactions would bring agents to a different interpretation. Table 2 gives an idea of how many opportunities one may have to study the different coupling processes through virtual exploration of possible alternative realities.

If parameters in the simulation change and, for example, we move the “proximity” – i.e., how wide is the attempt to couple with other resources in the room –, we may find different equilibria. Although a full analysis of the model is inappropriate in this paper, given the focus on the empirical case and the theoretical framework developed, it is possible to anticipate that a preliminary test of the conditions in this simulation seems to suggest that “proximity” significantly affects the end result. This is in line with the analysis above in that the ability to relate with other resources in the room brings individuals to showing a positive attitude toward a shared common activity. This can be interpreted, on the one hand, as a relaxation of one’s own assumption of roles and temporal understanding, and of a better match between distributed cognitive activities and what happens during the meeting, on the other hand.

Of course, more conditions need to be tested to fully support the qualitative findings. However, this preliminary test seems to suggest that distributed and ecological elements exemplified in CM1 and CM2 are essential to our understanding of organizational cognition.

A final note on the simulation concerns computationalism. In fact, using a computational tool such as an ABM may give the impression that we are back to a computationalist view of cognition. Nothing can be farthest from the truth. ABM are artificial representations of processes that may happen in the observable world. While their outcomes can be compared to observed data or to reason around a phenomenon, by no means their internal processes are to be intended as a 1:1 representation of the observed system. That would be a copy not a model of the observed. Having said that, ABMs have limitations (for more details see Secchi, 2022; Chapter 6). In fact, the subjective choices of the modeler affect quite significantly the way in which processes, parameters, mechanisms, etc. are simulated. Another limitation refers to selectivity; the ease with which ABM handles complexity may lead to models too difficult to analyze. Finally, even when results are insightful one should refrain from directly transferring results to practice and always remember these are indeed computational simulations.

## Conclusion

In this paper, we have argued that the dynamics of organizing should be understood and explained from the perspective of a distributed and ecological idea of cognition, which enables us to investigate organizing as a cognitive process emerging from the interactions among elements in a dynamic system. We conceived of *organizational cognition* in terms of organizing, whereby we contribute to research from a process-based philosophy on organizations (e.g., Langley and Tsoukas, 2010; Hernes, 2014). Also, we find important similarities with recent developments within the sensemaking literature (e.g., Weick, 1995; Cunliffe et al., 2004; Orlikowski, 2007; Whiteman and Cooper, 2011; Cunliffe and Coupland, 2012) with more embodied and embedded views on sensemaking, which includes sociomaterial, temporal and ecological aspects. However, the move toward a distributed and ecological perspective suggests that the unit of analysis shifts from the individual to the system, i.e., the interrelation between bodies and environmental structures, which allows us to analyze organizations in terms of interconnected cognitive resources, or, ecologies (Chemero, 2011; Steffensen, 2011). Conceptualizing cognition in terms of “enabling conditions” (Cowley et al., 2017), we see organizing as a process that is enabled by a number of coupling mechanisms put in place to get people attuned with the process. Following this approach, we studied the elements of a distributed and ecological approach, taking a specific case as an explanatory example.

In the paper, we focus our analysis on the Ø-Hop Case where something very typical in the life of business organizations happen. And yet, we were able to isolate specific cognitive trajectories and identify how interactional patterns were distributed on and constrained by different timescales to influence thinking and behavior in the group. The theory presented uses two different types of cognitive mechanisms – i.e., “socio-material” (or CM1) and “conceptual” (or CM2) — to operationalize the concepts and apply them to a qualitative interactional analysis of video material and to a computational simulation.

We found that the individuals had different understandings about what the activity meant, and the division between employees and management became increasingly apparent through the meeting, not just based on unequal power positions, but grounded in different conceptual and socio/material realizations. For various reasons, this difference created a breeding ground for tension between sub-groups, a tension that formed the trajectory of the meeting. This was observable on the video (and in the two sequences in Boxes 1, 2), when individuals within each sub-group interacted and tended to co-adapt to each other.

Further, the Ø-Hop Case read by the lenses of our framework allowed us to create an agent-based computational simulation. With relatively few assumptions for this model, we were able to demonstrate that the dynamic of the Case is a special case of similar conditions where individuals meet to discuss at a meeting, for example. This means that the framework (the model) is a useful way, not only to describe cognitive processes in organizations, but also to study counterfactuals (what-ifs). The ABM also points at the fact that the framework is particularly suited to describe complex adaptive systems (Miller and Page, 2007) - such as those in the realm of organizational cognition.

We suggest, that understanding human action from a distributed and ecological perspective may contribute to a deeper understanding of organizational cognition and, in the same vein, shed new light on the way in which organizing is accomplished as a joint activity. Under these lenses, organizations can be viewed as distributed networks of thoughts and actions that takes into consideration how groups of people generate output as they use each other as well as material artifacts as cognitive resources in natural settings (Trasmundi, 2020).

This article contributes to the literature in two distinct and complementary ways. On the one hand, it proposes a distributed and ecological approach to the study of organizational cognition. This is something that is long overdue, given the developments among cognitive science that took place since the Nineties. On the other hand, we have shown how this approach can be operationalized in organizational contexts, with a framework and a computational simulation.

## Data Availability Statement

The original contributions presented in the study are included in the article/Supplementary Material, further inquiries can be directed to the corresponding author/s.

## Ethics Statement

Ethical review and approval were not required for the study on human participants in accordance with the local legislation and institutional requirements. The participants provided their written informed consent to participate in this study.

## Author Contributions

All authors listed have made a substantial, direct, and intellectual contribution to the work, and approved it for publication.

## Conflict of Interest

The authors declare that the research was conducted in the absence of any commercial or financial relationships that could be construed as a potential conflict of interest.

## Publisher’s Note

All claims expressed in this article are solely those of the authors and do not necessarily represent those of their affiliated organizations, or those of the publisher, the editors and the reviewers. Any product that may be evaluated in this article, or claim that may be made by its manufacturer, is not guaranteed or endorsed by the publisher.

## Footnotes

1 We refer to a not better specified “group” to indicate that these mechanisms can apply to teams as well as various types of formal or informal organizations.
