# Supplementary material for: A Distributed Framework for the Study of Organizational Cognition in Meetings
Source: Front Psychol. 2022 May 18;13:769007. doi: 10.3389/fpsyg.2022.769007 (PMC9159498; doi:10.3389/fpsyg.2022.769007)
Supplement: Supplementary file 1 [file Data_Sheet_1.zip › Supplementary-Materials-CMM.pdf]

The `CMM 2.1.0` Model

Supplementary Materials

Davide Secchi

Research Centre for Computational & Organisational Cognition

University of Southern Denmark

April 21, 2022

This document presents the `CMM 2.1.0` Model in connection with the publication Jensen, Astrid, Secchi, Davide, and Wiben Jensen, Thomas, "A distributed framework for the study of organizational cognition", *Frontiers of Psychology*[1]. This file contains a description of the model that follows the ODD (Overview, Design, and Details) protocol to report Agent-Based Models. Additional information on the model can also be found on the `Info` tab in the `NetLogo` Model (uploaded separately). This document may be updated in the future. For up to date documentation on this model please check the OpenABM website at https://www.comses.net/codebases/.

Parts of this document are standard notes that I include in most of files serving similar purposes.

CONTENTS

1 The ODD Protocol **3**

1.1 Overview . . . 3

1.1.1 Purpose and pattern . . . 3

1.1.2 Entities, state variables, and scales . . . 4

1.1.3 Process overview and scheduling . . . 5

1.2 Design concepts . . . 8

1.2.1 Emergence . . . 8

1.2.2 Adaptation . . . 8

1.2.3 Objectives . . . 9

[1]When I am preparing the materials, the article has been accepted but its final version is still yet to be available on the website; https://www.frontiersin.org/articles/10.3389/fpsyg.2022.769007/abstract.

1.2.4 Learning . . . 9
1.2.5 Prediction . . . 9
1.2.6 Sensing . . . 10
1.2.7 Interaction . . . 10
1.2.8 Stochasticity . . . 10
1.2.9 Collectives . . . 11
1.2.10 Observation . . . 11
1.3 Details . . . 11
1.3.1 Initialization . . . 12
1.3.2 Input data . . . 12
1.3.3 Submodels . . . 12

2 What's Next **13**

References **14**

1 THE ODD PROTOCOL

The ODD Protocol is a standard introduced to help modelers describe their ABM by following a threefold structure: Overview, Design, and Details (Grimm et al., 2017). First introduced to suit ecology models, it has been updated several times in the last decade (Polhill, 2010; Grimm et al., 2010) until a latest update appeared in the *Journal of Artificial Societies and Social Simulation* (JASSS) in 2020 (Grimm et al., 2020).

The version of the model that has been uploaded on the platform is the same that is referred to in the publication Jensen et al. (2022).

The following pages cover all the sections in which the ODD protocol is specified.

1.1 Overview

This Subsection is dedicated to providing readers with a general understanding of the model, outlining its purpose, describing the agents, and sketching the process.

1.1.1 Purpose and pattern

`CMM 2.1.0` is a simulation that has an illustrative purpose. This is to demonstrate how a cognitive process works in a meeting, while participants try to discuss the points in the agenda. In capturing ecological aspects of embodied, distributed, and extended (EDEC, as per Secchi, 2021b) perspectives, it wants to reproduce how cognition happens in a business meeting.

It splits cognition's basic components and studies them as

(a) *socio-material* couplings (CM1)—the way in which participants interact with other participants and material resources;

(b) *conceptual* couplings (CM2)—interactions with the group as a whole, with ideas, procedures, and time.

According to Edmonds et al. (2019), this model fits into the *illustration* of how an EDEC system works. What makes the model particularly interesting is the fact that these perspectives to cognition have been criticized as being too broad, closer to philosophical approaches rather than to testable propositions (Miłkowski et al., 2018). By isolating the mechanisms at work in the procedures of this model, the simulation shows that EDEC is more than a generic approach. However, this is one of the few times this has been attempted (see, for example, the model described in Secchi, 2021b,a).

How does the model accomplish this aim? `CMM 2.1.0` splits cognition in several processes. By taking a qualitative discourse analysis of a meeting (see details in the actual paper Jensen et al., 2022) as input for parametrization, the model isolates different mechanisms—ie ways for the various parts of the cognitive system to connect and interact. These are designed to include

most (if not all) possible connections, among people, and between people and ideas, artifacts, etc. More on this in the pages below.

The first intention of the model is to replicate findings from the qualitative analysis. This means that the general dynamic—ie understanding, content divergence/convergence, idea of the group (identification), and role of procedures.[2] The main pattern is that of showing divergence between employees and management as well as gradual understanding, given the initial configuration of parameters calibrated to mimic the empirical data from the actual meeting. Taking this as a baseline case, the model then can be used to understand which factors lead to such an outcome, thus defining EDEC cognitive enablers/disablers.

1.1.2 Entities, state variables, and scales

There are three agent-types in this model: (a) employees, (b) managers, and (c) a 'talk.' The first two are intuitive enough, given the context is a meeting with participants. The third agent type is the act of speaking at the meeting. The idea of using a separate agent to frame the act of talking derives from an extended cognitive framing, where words/language are no more bound to the person who speaks, once words are spoken (Clark and Chalmers, 1998; Cowley, 2011).

The total number of agent-employees and -management is set to 9. One could determine the number of agent-managers with the parameter `num_managers` $[0, 9]$.

Another 'entity' that can be mentioned here is the environment. This is a meeting room, designed to replicate—in a very abstract way—the elements of the actual room where the meeting was held. In the center is a rectangular table, with notepads in front of every agent-person, and a videocamera placed outside of it in the bottom part of the environment, and a screen where slides and other materials are projected. In the upper part of the room one can also find a whiteboard, used to draw or write. The environment is static—ie there are no changes other than some participants moving to the screen or to the board.

Table 1 presents the three agent-types by giving a short recap of their characteristics and how they are parametrized.[3]

While the two 'people' agents are characterized by random variables, the behavior of which is controlled by the parameters specified in the upper part of Table 1, the agent-talk is only defined by the variable 'content' and, initially, by the parameter `content_level`.

[2] The four plots in the NetLogo Interface can be used as a first assessment of findings/patterns.

[3] A quick note should go here on a sightly different use of terminology. According to the support documentation for the ODD protocol (https://www.jasss.org/23/2/7/S1-ODD.pdf), any value that is attributed to the entities to distinguish between them is a *state variable*, while parameters are constants that usually appear in equations. In this document, we call parameters all those values that can be controlled by the modeler and are set at the beginning of the simulation. In other words, the parameters can be manipulated to perform the simulation. State variables, instead, are also set at the beginning of the simulation, but they cannot be controlled directly. If they can, then they can be considered parameters. Some parameters affect state variables directly, but I would distinguish between the calculated variables and the (constant) parameter value set at the beginning of the simulation.

Table 1: Parameter Notations and Values

Parameter	Notation	Type	Values	Description
Setup conditions				
Steps	s	S	100	Time in the simulation; otherwise stated, the number of opportunities that agents have to connect and interact.
Proximity	p	P	$[10, 20]$	This is the distance that offers agents the opportunity to connect. It can be seen as the screening range of sight each one in the room has.
Number of managers	N_m	P	$[0, 9]$	The number of managers in the room. Nine is the max number of participants and this means that one could perform the simulation in a meeting where only managers interact.
Understanding Threshold	T_u	$[0, 1]$	P	A global threshold that defines how much understanding is needed to actually interpret content correctly (i.e. in line with the others).
Procedures Threshold	T_ϕ	$[0, 1]$	P	A global threshold that defines what it takes to be aware of meeting (organizational) procedures.
Experience Threshold	T_ϵ	$[0, 1]$	P	A global threshold that defines how much experience is necessary to affect the meeting outcome.
Content	C	$[0, 1]$	P	The level to which the agent-talk sets its content at the beginning (i.e. at $s = 2$).
Agent-employees and -managers				
Hierarchy	H	$\sim \mathcal{N}(0, 1)$	SV	This is the extent to which hierarchy is valued by the agent. Positive values indicate the agent puts a higher weight on actions from a manager. It affects both $CM1_i$ and $CM1_ii$.
Group attunement	G	$\sim \mathcal{N}(1, 1)$	SV	This variable regulates the extent to which $CM2_i$ enters into play. High attunement with the group leads to a better understanding of others (controlled by `proximity`).
Understanding	U	$\sim \mathcal{U}(0, 1)$	SV	It is the extend to which agents interpret what is said by others and understand it—regulated by the threshold (T_u) as indicated above.
Procedures	ϕ	$\sim \mathcal{U}(0, 1)$	SV	The uniform distribution indicates how much agents are aware and respect procedures—i.e. the typical "rules of the game" in a meeting. It is regulated by its threshold T_ϕ as indicated above
Acquaint	α	$\sim \mathcal{N}(0, 1)$	SV	When two agents show positive values of this variable they know each other, perhaps from working together in the same team or simply by being friends outside of work.
Experience	ϵ	$\sim \mathcal{U}(0, 1)$	SV	The amount of knowledge deriving from seniority (tenure) in the organization and from having been for longer in positions similar to the current one. Experience enters into effect according to the threshold T_ϵ, described above.

SV: state variable; P: parameter; S = scale.

1.1.3 Process overview and scheduling

There are a number of processes in the simulation, roughly one per coupling mechanism. There are two coupling mechanisms under $CM1$ and four under $CM2$:

- socio-material couplings ($CM1$)
  - people ($CM1_i$)—interactions among participants, mainly affected by talking;
  - material ($CM1_{ii}$)—interactions with the artifacts in the room (e.g., notepad, board);
- conceptual couplings ($CM2$)
  - idea of the group/organization ($CM2_i$)—interactions with the idea of the group in the room (for example, how one identifies with it);

- topic ($CM2_{ii}$)—interactions and understanding of the points under discussion during the meeting;
- procedures ($CM2_{iii}$)—the extent to which one understands and agrees with the procedures in place (the "rule of the game" of the meeting);
- time ($CM2_{iv}$)—the idea each one of the participants have of time, defined on a fast/slow timescale.

CM1 Processes
The first process is to establish connections among agents. All agents take action by looking at their `hierarchy`. If $H > 0$ then a link with a manager in radius of `proximity` is established. Otherwise, a connection is established with an active artifact—i.e. those with operational variable `resources` > 0—in radius of `proximity`.

While the latter connection (with an artifact) leads to a further *activation* of the artifact, the former is important for the `talking` procedure (see below).

Continuing from the above, when a manager has a weak understanding of `hierarchy` (i.e. $H \leq 0$) then the one manager with $H_i = min(H)$ focuses on using an artifact rather than engaging with others in the room.

The activation of $CM1_i$ is performed by a process called `talking`. This initiates with the creation of an agent-talk (always when $s = 2$) that emerges from one of the managers. As the simulation progresses (for $s > 5$), agent-managers with the largest capability of understanding—i.e. $Ui = max(U_m)$—start talking. Further progression ($s > 10$) sees agent-employees involved as well, with those feeling to be misunderstanding something—i.e. $U < T_U$—and with a minimal understanding of procedures among employees—i.e. $P = min(P_e)$—start talking. It is important to notice here that managers talk if they believe they have a good understanding of the topic; employees, on the contrary, talk if they feel like they are not very much in line with the topic but have a decent attunement with the group ($G > 0$).

CM2 Processes
Employees with $G \leq 0$ are un-coupled, meaning that they do not connect with any artifact, anyone, or any abstraction. Their link die and these agents stop working on their data.

For employees with $G > 0$, they link with the agent-talk (they do not talk, but they listen) when $U_i > T_U$. When they do listen to a manager, their `understanding` re-calibrates by adding a value that is a function of the gap between the talking manager and themselves:

$$U_{e,t+1} = U_{e,t} + \mathcal{N}\left(U_{m,t} - U_{e,t}, \frac{|U_{m,t} - U_{e,t}|}{2}\right)$$

where $U_{e,t+1}$ is the `understanding` of the agent-employee at time $t + 1$, $U_{e,t}$ is the same at time t, and $U_{m,t}$ is `understanding` of the agent-manager connected to that agent-employee at time t. The expression $U_{m,t} - U_{e,t}$ is also known in this simulation as the gap.

If at the other end of the link there is no manager then the expression above changes as follows:

$$U_{e_1,t+1} = U_{e_1,t} + \frac{U_{e_2,t} - U_{e_1,t}}{10}$$

where the subscripts e_1 and e_2 are, respectively, employee and the one and at the other end of the link. The direction of understanding here can move up- or downwards as opposed to the one above, where the movement is always positive (upward). There is an increase in understanding when listening to a manager—that does not mean that the direction is the one that is actually useful. The other "correction" is more dynamic, hence it offers more chances to adapt (or fit, if you prefer that expression).

Managers follow a process very similar to the one described above. If a manager is sinked to another manager, then

$$U_{m_1,t+1} = U_{m_1,t} + \mathcal{U}\left(\frac{U_{m_2,t} - U_{m_1,t}}{10}\right)$$

where, similarly for the above, m_1 is one manager and m_2 is the other. When connected to an employee, then the expression becomes:

$$U_{m,t+1} = U_{m,t} + \frac{U_{e,t} - U_{m,t}}{100}.$$

Agents that are uncoupled re-calibrate their G to a random value that differs from the expression used at the beginning of the simulation (see Table 1)—this time they use $\mathcal{U}(0, 1)$. This is done to keep them out of interactions.

Dynamic processes

If two employees are acquainted, that is $\alpha > 0$, they become connected and adjust their interpretation of the talk (`content`) at a rate of $\rho = 0.02$, such that there is a slow progression towards the one of the two that generated the connection in the first place.

Also, the variable `procedures` changes its state. When $\phi_i > T_\phi$ then

$$G_{i,t+1} = G_{i,t} + \mathcal{U}\left(0, \frac{\phi}{2}\right)$$

where `group attunement` of an agent i at time $t+1$ is a function of its value at time t and modifies using a uniform distribution $\mathcal{U}$ with bounds 0 and $\frac{\phi}{2}$. The logic here is that one increases understanding of what the group is there for when procedures are well understood.

Continuing on the above, if $\phi > T_\phi$ and $G_i > 1$ then the formula above sees a reduction of G_i that is proportional to the knowledge of procedures.

Another factor to consider is `experience`. When $\epsilon_i > T_\epsilon$, then knowledge of procedures is enhanced:

$$\phi_{i,t+1} = \phi_{i,t} + \mathcal{U}\left(0, \frac{\epsilon}{4}\right)$$

and, similarly to the above, when $\phi_i > 1$ then there is a contraction (fluctuation) of this knowledge. The idea here is that procedures understanding increases (or modifies) as experience kicks in.

`Content` for agent-employees and agent-managers are the simple sum of the links they have with artifacts and other agents (the actual value is expressed in a $[0, 1]$ form). The way in which `content` adjusts itself is through the agent-talk. The difference $\Delta = C_i - C_T$—i.e. content in the agent-employee or -manager and that in the agent-talk—is used to create a random value that

is $\mathcal{N}\left(\frac{\Delta/100}{|\Delta/100|}\right)$. Then, if $C_T > 1$ then it decreases of a fraction made of $\mathcal{U}(0, 0.25)$, while it increases of the same fraction if otherwise.

1.2 Design concepts

The basic concepts that inspire `CMM 2.1.0` have been already mentioned above. The EDEC perspective is one of them (Varela et al., 1991; Hutchins, 1995; Clark and Chalmers, 1998) in that the *coupling* mechanisms are derived directly from it. A more articulate detail on why and how EDEC is relevant for this model is offered in Jensen et al. (2022).

This model is only a first attempt to illustrate cognition under the EDEC perspective and it is particularly relevant because it focuses on the *social* aspects and, at the same time, on the *abstractions* that affect our thinking. Both the social and the abstract elements of cognition have been overlooked by the literature on cognition, let alone the more traditional computationalist/representationalist view. In taking EDEC on board, the model attempts to play with most of the mechanisms listed above. Given the width and depth of what is covered by EDEC—especially as it extends to social resources (Secchi, 2021b; Secchi and Bardone, 2009)—the model is intended as one of the first tentatives to codify them. Other attempts have been made, especially in Secchi's (2021b; 2016; 2011b; 2021a; 2020) and Secchi and Bardone's work (2009; 2013; 2017).

1.2.1 Emergence

By taking a classic definition of emergence (e.g., Cunningham, 2001, *epistemic emergence*$_2$), the behavior of the system cannot be fully tracked down to the characteristics of its parts. In this simulation, there are a number of surprising results that may qualify as emergent (Epstein, 1999).

The way in which the model shows convergent or divergent results is very difficult to trace back to any single parameter setting. This indicates that interactions among participants and available resources determine the end result, rather than a direct connection with initial conditions. This is in line with what theorized by Secchi and Cowley (2021) in that agent-based models are especially relevant for cognition in that they work on the basis of the *meso* domain. In organizational settings (such as the meeting), this can also be called *social organizing* (see also Secchi et al., 2022; Secchi, 2021b). Interconnections among cognitive resources make results uncertain and define what is the outcome of the meeting. Emergent is here tied to the concept of *social organizing*.

1.2.2 Adaptation

Much of what is described under the paragraph "dynamic processes" is very much tied to adaptation. In this model, agent characteristics adapt rather than agents as a whole. As seen above, `content`, `understanding`, and `procedures` adapt depending on particular interactions that agents have with other agents and the agent-talk. Rather than repeat the specific

conditions that lead to adaptive (dynamic) behavior, it is probably better to refer to what has been already written above.

1.2.3 Objectives

The objectives of the three adaptation mechanisms are rather straightforward:

- `content`—the goal is to adjust the interpretation of content based on interactions. As the initial setting C changes, its interpretation may become more or less straightforward for participants.
- `understanding`—this is a broader concept and it relates to attuning oneself to the others while they are talking. It is connected to `content` although the goal of its adjustments are more tied to social interactions rather than interaction with artifacts and immaterial resources.
- `procedures`—the goal of adjusting this item is simply to make participants understands the rules of the meeting more as time goes by. It is fair to assume that, even those who are not accustomed to meetings in a given organization, will soon learn how they work as the meeting progresses.

1.2.4 Learning

It is difficult to refer to 'learning' in a generic way, especially when the model deals with cognition. While there cannot be learning without cognitive activity, there could be cognitive activity without learning. `CMM 2.1.0` does not explicitly refer to learning but to the cognitive mechanisms (couplings) that are necessary for it to happen. All the three objectives above and their adaptations represent agents that learn (broadly speaking), how to deal with meeting procedures, content of the talk, and understanding of meeting dynamics. At the same time, the focus of the model is (again) not learning but the cognitive mechanisms that may allow someone to learn as well as perform other actions.

1.2.5 Prediction

The model is not set to predict meeting outcomes in general, just the extent to which participants converge in their understanding of what is going on. There are given configurations of parameters that will replicate the findings of the qualitative empirical research that has been used to develop the model in the first place. These conditions are:
$C = 0.51, T_U = .50, T_\epsilon = 0.75, T_\phi = 0.20,$ and $p = 11$. This can hardly be called prediction, but these settings reproduce the outcomes of the empirical data. Of course, there is a level of judgement in this, since the data is qualitative and there is a degree of interpretation that is necessary (the thorough analysis in Jensen et al., 2022, clarifies what the outcomes look like).

Whether `CMM 2.1.0` is capable of prediction cannot be known at the moment. The process would be that of gathering additional data from various meetings, and compare those results to

specific configurations of parameters. A more quantitative approach should be used in the empirical research and a more specifically defined outcome would be preferable.

1.2.6 Sensing

The entire model is based on the way in which agents "perceive" their surroundings, especially what derives from material, immaterial, and social cognitive resources. The dynamical procedures described above are about sensing—i.e. what agents "know" and what they think the other agents know. This own knowledge and perception of others' knowledge determines the way in which own knowledge changes. And this change is sometimes subject to pseudo-random adjustments (see above).

The entire process is based on an *extended rationality* concept (Secchi, 2011a). This is equivalent to a dynamic bounded rationality concept where the limits of cognition move depending on the resources available. The interpretation of content, situational understanding and procedures dexterity are examples of how individuals adapt to different contextual cues.

1.2.7 Interaction

`CMM 2.1.0` is built on the interaction among meeting participants, the artifacts around them and the immaterial (ideal) resources. The processes above describe the way in which these three types of resources interact and determine adaptation and change.

More specifically, the model visualizes interactions through colored links. The different color depends on the type of resource to which participants connect, hence it tells something about the type of coupling mechanism. These are directed links, even though the dynamic is, in some cases, two-way—e.g., both participants' understanding, if connected, may be affected by the interaction.

1.2.8 Stochasticity

Random components in this simulation are:

- Initial attribution of an agent's characteristics—all state variables are based on pseudo-random distributions, either normal or uniform:
  - $H \sim \mathcal{N}(0, 1)$
  - $G \sim \mathcal{N}(1, 1)$
  - $U \sim \mathcal{U}(0, 1)$
  - $\phi \sim \mathcal{U}(0, 1)$
  - $\alpha \sim \mathcal{N}(0, 1)$
  - $\epsilon \sim \mathcal{U}(0, 1)$

- When employees are connected to a manager, the way in which `understanding` (U) updates has a random normal component, such that both size and sign of the change vary unpredictably.
- Similarly to the above, when a manager is linked to another manager, their `understanding` (U) changes based on a uniform distribution between zero and the size of the distance between the two agents' understanding.
- `Procedures` (ϕ) affect the way in which `group attunement` (G) updates, calculated on $\mathcal{U}(0, \phi/2)$ (see above).
- Similarly, ϕ updates using a random component calculated on ϵ, $\mathcal{U}(0, \epsilon/4)$ (see above).
- Also `content` (C) changes based on a random-normal component (see the formula above).

1.2.9 Collectives

The group that meets in the room is the collective of `CMM 2.1.0`. The behavior of the collective is what this model analyzes, even if the cumulative behavior is an aggregation of individual action.

1.2.10 Observation

The information collected from the model—what can be called *outcome variables*—is visualized in the four plots on the Interface. All values are calculated at every step:

- `content`—mean content of employees $\bar{C_e}$, mean content of managers $\bar{C_m}$, and content of the talk C_t.
- `group attunement`—simple value dynamic calculated for each agent G_i.
- `procedures`—simple value dynamic calculated for each agent ϕ_i.
- `understanding`—mean understanding for employees U_e, mean understanding for managers ϕ_m.

1.3 Details

This part of the ODD protocol is necessarily slim, because of the purpose of the `CMM 2.1.0`. In fact, the model serves an *illustration* purpose and there is no input data.

1.3.1 Initialization

Before hitting the 'setup' button, the model needs most parameters to be specified through the sliders on the Interface. By clicking the button, the layout of the room appears as well as the agents (all but the agent-talk). The parameters that need to be determined are (see also Table 1):

- `proximity` p;
- `content_level` C;
- `num_managers` N_m;
- `understanding_threshold` T_U;
- `procedures_threshold` T_ϕ;
- `experience_threshold` T_ϵ;

When the 'start' button is clicked the agents start connecting and interacting with each other, depending on their characteristics. There is no more input required from the user.

1.3.2 Input data

The model does not uses any input data.

1.3.3 Submodels

`CMM 2.1.0` is relatively simple, although it has a few sections of its code that can be considered as *blocks* rather than submodels. I am writing this because none of these portions of code work in isolation, but needs the others to function properly. These modeling *blocks* are:

- $CM1_i$—interactions with artifacts;
- $CM1_ii$—interactions with agent-people;
- $CM2_i$—interactions with ideas (G);
- $CM2_ii$—interactions with the topic (U);
- $CM2_iii$—interactions with procedures (ϕ);
- $CM2_iv$—interactions with time (dynamics through s);

2 WHAT'S NEXT

There are at least two points of interest of this model. One is that this is one of the very few attempts to build a model based on a strict relation with qualitative data. There is a debate on how to build a methodology for implementing qualitative data onto ABM on a systematic basis (see, for example, the special issue in JASSS Edmonds, 2015). `CMM 2.1.0` can be considered as a first attempt to connect these two domains by using video analysis through discourse analysis. The model presents elements that have emerged through that analysis. Although these elements appear in an order that may differ from those of the qualitative analysis, the cognitive-bound outcomes are very similar. The data informed mainly the parameters and the procedures in this model, rather than the values of the parameters. More needs to be done on this respect, to understand how to systematically build models out of qualitative evidence.

A second point of interest is that this model could be developed further to (a) strengthen its ties to the six mechanisms and (b) provide a better generalization of the processes involved when cognition is considered during meetings. This means that a full calibration of the model needs to be performed through sensitivity analysis, to define how outcomes may vary depending on parameter values. This, in turn, would require the thorough examination of simulation data that is, at the moment, yet to be performed.

REFERENCES

Bardone, E. and Secchi, D. (2017). Inquisitiveness: Distributing rational thinking. *Team Performance Management*, 23(1/2):66–81.

Clark, A. and Chalmers, D. J. (1998). The extended mind. *Analysis*, 58:7–19.

Cowley, S. J., editor (2011). *Distributed Language*. Amsterdam: Benjamins.

Cunningham, B. (2001). The reemergence of ‘emergence’. *Philosophy of Science*, 68:S62–S75.

Edmonds, B. (2015). Using qualitative evidence to inform the specification of agent-based models. *Journal of Artificial Societies and Social Simulation*, 18(1):18.

Edmonds, B., Page, C. L., Bithell, M., Chattoe-Brown, E., Grimm, V., Meyer, R., Montanola-Sales, C., Ormerod, P., Root, H., and Squazzoni, F. (2019). Different modelling purposes. *Journal of artificial societies and social simulation*, 22(3):6.

Epstein, J. M. (1999). Agent-based computational models and generative social science. *Complexity*, 4(5):41–57.

Grimm, V., Berger, U., DeAngelis, D. L., Polhill, J. G., Giske, J., and Railsback, S. F. (2010). The odd protocol: a review and first update. *Ecological modelling*, 221(23):2760–2768.

Grimm, V., Polhill, G., and Touza, J. (2017). Documenting social simulation models: The ODD protocol as a standard. In Edmonds, B. and Meyer, R., editors, *Simulating Social Complexity. A Handbook*, pages 349–365. Springer, Cham.

Grimm, V., Railsback, S. F., Vincenot, C. E., Berger, U., Gallagher, C., DeAngelis, D. L., Edmonds, B., Ge, J., Giske, J., Groeneveld, J., et al. (2020). The ODD protocol for describing agent-based and other simulation models: A second update to improve clarity, replication, and structural realism. *Journal of Artificial Societies and Social Simulation*, 23(2).

Hutchins, E. (1995). *Cognition in the wild*. Cambridge, MA: MIT Press.

Jensen, A., Secchi, D., and Jensen, T. W. (2022). A distributed framework for the study of organizational cognition: The meeting. *Frontiers in Psychology – Organizational Psychology*, forthcoming.

Miłkowski, M., Clowes, R., Rucińska, Z., Przegalińska, A., Zawidzki, T., Krueger, J., Gies, A., McGann, M., Afeltowicz, L., Wachowski, W., et al. (2018). From wide cognition to mechanisms: A silent revolution. *Frontiers in Psychology*, 9:2393.

Polhill, J. G. (2010). ODD updated. *Journal of Artificial Societies and Social Simulation*, 13(4):9.

Secchi, D. (2011a). Banks and discrimination: How far can we go with competition? A reply to Block, Snow, and Stringham. *Business and Society Review*, 116(1):53–83.

Secchi, D. (2011b). *Extendable rationality. Understanding decision making in organizations*. New York: Springer.

Secchi, D. (2016). Boundary conditions for the emergence of 'docility:' An agent-based model and simulation. In Secchi, D. and Neumann, M., editors, *Agent-Based Simulation of Organizational Behavior. New Frontiers of Social Science Research*, pages 175–200. New York: Springer.

Secchi, D. (2020). A typology of non-functional information. In Stephanidis, C., Harris, D., Li, W.-C., Schmorrow, D. D., Fidopiastis, C. M., Zaphiris, P., Ioannou, A., Sottilare, X. F. R. A., and Schwarz, J., editors, *HCI International 2020 - Late Breaking Papers: Cognition, Learning and Games*, volume 12425 of *Lecture Notes in Computer Science*, pages 240–254. Springer Nature.

Secchi, D. (2021a). Cognitive attunement in the face of organizational plasticity. *Evidence-Based Human Resource Management*, 9(2):192–208.

Secchi, D. (2021b). *Computational Organizational Cognition. A study on thinking and action in organizations*. Emerald Publishing.

Secchi, D. and Bardone, E. (2009). Super-docility in organizations. An evolutionary model. *International Journal of Organization Theory and Behavior*, 12(3):339–379.

Secchi, D. and Bardone, E. (2013). Socially distributed cognition and intra-organizational bandwagons: Theoretical framework, model, and simulation. *International Journal of Organization Theory and Behavior*, 16(4):521–572.

Secchi, D. and Cowley, S. J. (2021). Organisational cognition: What it is and how it works. *European Management Review*, 18(2):79–92.

Secchi, D., Gahrn-Andersen, R., and Cowley, S. J., editors (2022). *Organizational Cognition: The Theory of Social Organizing*. Routledge (forthcoming).

Varela, F. J., Thompson, E., and Rosch, E. (1991). *The Embodied Mind: Cognitive Science and Human Experience*. Cambridge, MA: MIT Press.
